# Supplementary material for: The causal role of circulating inflammatory markers in osteoporosis: a bidirectional Mendelian randomized study
Source: Front Immunol. 2024 Jul 18;15:1412298. doi: 10.3389/fimmu.2024.1412298 (PMC11291241; doi:10.3389/fimmu.2024.1412298)
Supplement: Supplementary file 1 [file DataSheet_1.zip › supplementary material/OSTEOPOROSIS-CIM/GCST90274840.tsv.gz_forest.pdf]

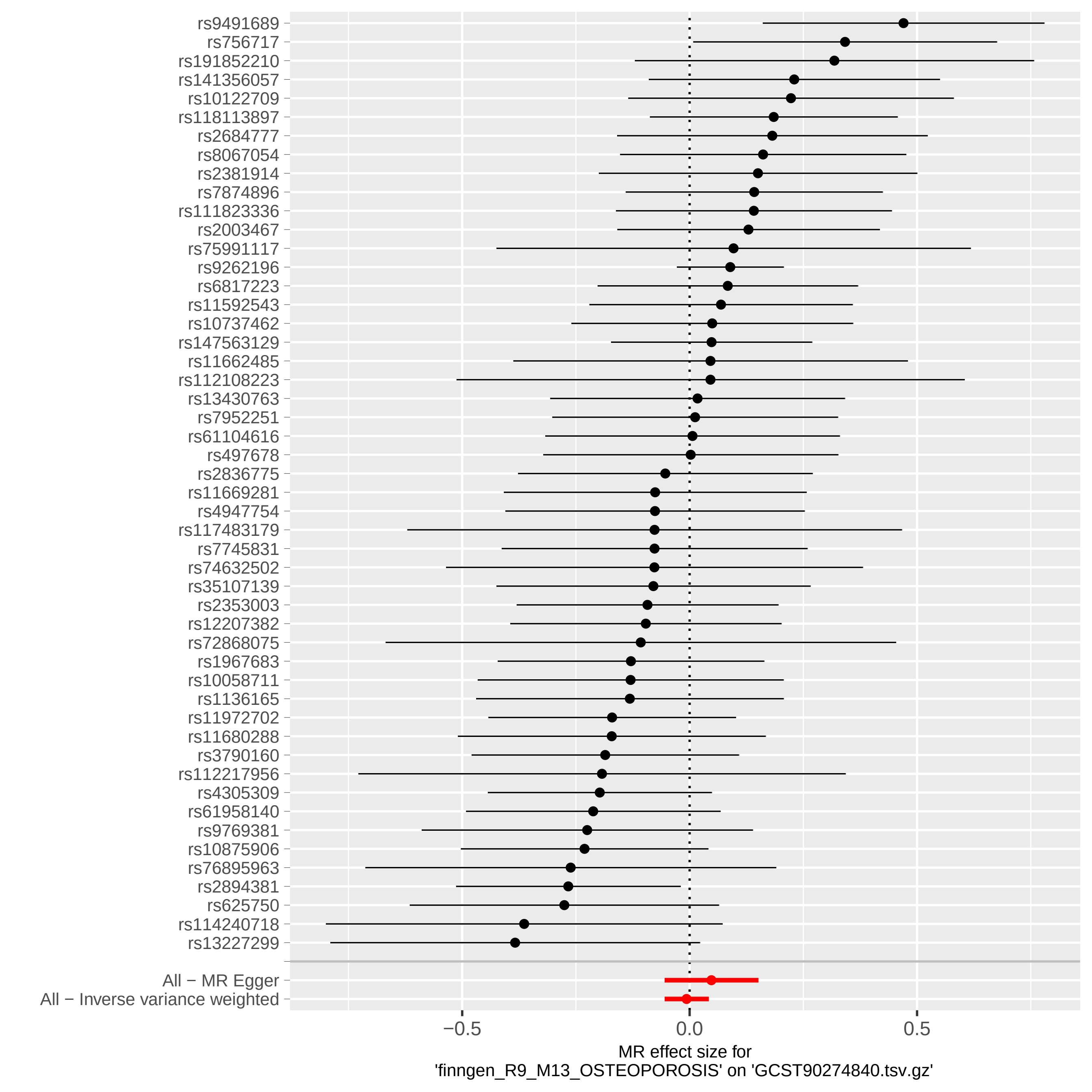
rs9491689
rs756717
rs191852210
rs141356057
rs10122709
rs118113897
rs2684777
rs8067054
rs2381914
rs7874896
rs111823336
rs2003467
rs75991117
rs9262196
rs6817223
rs11592543
rs10737462
rs147563129
rs11662485
rs112108223
rs13430763
rs7952251
rs61104616
rs497678
rs2836775
rs11669281
rs4947754
rs117483179
rs7745831
rs74632502
rs35107139
rs2353003
rs12207382
rs72868075
rs1967683
rs10058711
rs1136165
rs11972702
rs11680288
rs3790160
rs112217956
rs4305309
rs61958140
rs9769381
rs10875906
rs76895963
rs2894381
rs625750
rs114240718
rs13227299
All − MR Egger
All − Inverse variance weighted
−0.5
0.0
0.5
MR effect size for
'finngen_R9_M13_OSTEOPOROSIS' on 'GCST90274840.tsv.gz'
